# Chronic Exposure to Arsenic in Drinking Water Causes Alterations in Locomotor Activity and Decreases Striatal mRNA for the D2 Dopamine Receptor in CD1 Male Mice

**DOI:** 10.1155/2016/4763434

**Published:** 2016-06-08

**Authors:** Claudia Leticia Moreno Ávila, Jorge H. Limón-Pacheco, Magda Giordano, Verónica M. Rodríguez

**Affiliations:** Departamento de Neurobiología Conductual y Cognitiva, Instituto de Neurobiología, Universidad Nacional Autónoma de México, Boulevard Juriquilla 3001, 76230 Querétaro, QRO, Mexico

## Abstract

Arsenic exposure has been associated with sensory, motor, memory, and learning alterations in humans and alterations in locomotor activity, behavioral tasks, and neurotransmitters systems in rodents. In this study, CD1 mice were exposed to 0.5 or 5.0 mg As/L of drinking water for 6 months. Locomotor activity, aggression, interspecific behavior and physical appearance, monoamines levels, and expression of the messenger for dopamine receptors D1 and D2 were assessed. Arsenic exposure produced hypoactivity at six months and other behaviors such as rearing and on-wall rearing and barbering showed both increases and decreases. No alterations on aggressive behavior or monoamines levels in striatum or frontal cortex were observed. A significant decrease in the expression of mRNA for D2 receptors was found in striatum of mice exposed to 5.0 mg As/L. This study provides evidence for the use of dopamine receptor D2 as potential target of arsenic toxicity in the dopaminergic system.

## 1. Introduction

Arsenic (As) is a natural occurring element widely found in the environment, due to its ubiquitous presence in the earth's crust and to its high usage in several anthropogenic activities such as fabrication of computer chips, glass manufacturing, mining waste, agrochemicals (insecticides, rodenticides, herbicides, plant desiccants, and fertilizers), and wood preservatives [1]. The main route of As exposure is via drinking water (DW), which in several regions of the world exceeds the World Health Organization (WHO) permitted level of 0.010 mg As/L. For example, levels as high as 0.2 mg As/L were found in Argentina [2], 2.97 mg As/L in Bangladesh [3], 0.5 mg As/L in Chile [4], 0.4 mg As/L in Mexico [5], and 0.8 mg As/L in Taiwan [6]. There are also reports of accidental exposure through drinks or foods tainted with As. An example of such events was the ingestion of contaminated milk powder produced by the Morinaga Milk Industry Company in Japan during the 1950s, when infants were exposed to doses of 4.2–7.0 mg As/L of milk [7].

Ingestion of DW contaminated with As is associated with several adverse effects on human health [8–15]. Effects on the central nervous system include memory deficits [11, 16], reduced intellectual functions (decreased verbal IQ) [12], epilepsy, minimal brain damage, mental retardation, and IQ less than 85 [7], and mood changes including depression, easy irritability, anxiety disorder, or lack of concentration [17].

Behavioral studies have primarily used the rat model of As exposure and have found that locomotor activity is altered depending on the dose of As administered, route and time of exposure, and specific strain used [18]. Studies using the mouse as a model have also found changes in locomotor activity, both increases and decreases, related to dose, duration of exposure, strain, and gender [19, 20]. These and other studies also assessed the effects of As exposure on the dopaminergic system and have found that As exposure either reduced striatal levels of the dopamine (DA) metabolites [19], increased DA content in striatal homogenates [21], or decreased striatal DA in female mice in a dose-dependent manner [20].

One of the advantages of using mice instead of rats is that mouse red blood cells do not sequester As by binding it to the sulfhydryl groups of hemoglobin as the rat does, which explains why the rat is not a good toxicokinetic model of As exposure [22–24]. In addition, the availability of transgenic mouse strains could contribute to understanding the mechanisms of action of As. However, mouse strains show substantial behavioral variability, and as Adams et al. suggest [25] the choice of host strain in transgenic research must be made carefully. Outbred strains like the CD1 are robust, easy to breed, and resistant to diseases, mimic genetically heterogenous populations [25], and exhibit a more variable phenotype [26]. In contrast, “inbred strains like the C57Bl/6J are considered to be nearly homozygous (genetically identical) and are usually chosen for their relatively restricted genetic variability and reliable behavioral profile” [26]. Also, earlier studies suggest that the C57Bl/6 strain may have lowered dopaminergic function, as evidenced by its increased susceptibility to the effects of haloperidol and to the neurotoxin MPTP [27].

Previous studies from our laboratory and from others have demonstrated that the dopaminergic system is a target of As toxicity altering its functions at several levels of regulation including DA synthesis and signaling. These include disturbance in DA levels in a gender-specific manner, accompanied by changes in mRNA expression of genes related to dopaminergic and antioxidant systems in the striatum of different rodent models such as DA receptors D1, D2, D3, and D4 [20, 28, 29]. According to those studies DA receptors seem to be good candidates to evaluate alterations caused by chronic ingestion of As via drinking water in the dopaminergic system in rodents. To support its potential use as biomarkers, the expression of DA receptors D1a and D2 was evaluated together with the locomotor activity and aggressive behavior, levels of DA, and its metabolites in striatal tissue of CD1 mice exposed to 0.5 and 5.0 mg As/L of DW for six months. Then, we compared the results with those of previous studies using C57BL/6J mice, an inbred strain, and those described in rats, and discuss the potential use of DA receptor D2 as a potential biomarker of As toxicity in the dopaminergic system.

## 2. Experimental Design

### 2.1. Animals

Forty-five, two-month-old male CD1 mice were acquired from the vivarium of the Instituto de Neurobiologia, UNAM and kept under a 12-hour inverted dark/light cycle (lights on at 20:00) with constant temperature (23 ± 2°C). Experiments were carried out according to the Norma Oficial Mexicana de la Secretaría de Agricultura (SAGARPA NOM-062-ZOO-1999), which complies with the guidelines in the Institutional Animal Care and Use Committee Guidebook (NIH Publication 80-23, Bethesda, MD, USA, 1996), and were approved by the local committee on Bioethics.

### 2.2. Chemicals

Sodium arsenite (99.6% purity) was acquired from J.T. Baker (Phillipsburg, NJ, USA); reagents for high performance liquid chromatography with electrochemical detection (HPLC-ED) were acquired from Sigma-Aldrich (St. Louis, MO, USA), unless otherwise is stated. Of the inorganic As compounds, sodium arsenite is one of the most common trivalent compounds used in toxicological studies and resembles the presence of this form in wells of contaminated areas.

### 2.3. Materials and Methods

Fifteen mice per group received 0.5 or 5.0 mg As/L of DW for six months. The As-containing DW solutions were prepared daily from a 1000 mg As/L solution in deionized water, and the pH was adjusted to 7.0 in order to minimize the oxidation of arsenite to arsenate. Control groups received deionized water adjusted to pH = 7.0. Three separate groups of animals were used, and intermediate doses of As were chosen based on the results obtained in mice [20] or rats [30–32]. In order to achieve comparable levels of As in humans, mice have to be exposed to greater concentrations than those found in the environment. In this respect, mice metabolize and clear As and its metabolites from tissues more efficiently than humans (for more details, see [33]).

Body weight and the presence of body lesions and those not classified as lesions (whisker trimming, hair barbering, and disheveled coat) were evaluated weekly throughout the duration of the experiment. The locomotor activity, the presence of aggressive behaviors, and typical rodent behaviors such as rearing and grooming were evaluated monthly from the first to the sixth month of As exposure. After six months of exposure, mice were euthanized by cervical decapitation, brain was extracted, and both left and right striatum and frontal cortex were dissected on ice and frozen at −80°C; striatum and frontal cortex from one hemisphere were used to measure DA and serotonin and their metabolites, while the striatum from the other hemisphere was used to evaluate the expression of the genes for DA receptors (*Drd1* and* Drd2*).

### 2.4. Behavioral Tests

#### 2.4.1. Spontaneous Locomotor Activity

Once a month mice were individually placed in an automated locomotor activity chamber equipped with horizontal and vertical infrared beams (Accuscan Instruments Inc., Columbus, OH, USA). Locomotor activity was recorded, and data were collected over the course of a 25-hour session. The first hour, which is usually when the highest activity is displayed, was evaluated separately, form the remaining 24 h (12 h light : 12 h dark). The locomotor activity parameters evaluated included total distance (the distance in cm traveled by the animal) and horizontal activity (activity that blocks sensors on the chamber's horizontal axis). Food and water were available* ad libitum* during this session.

#### 2.4.2. On- and Off-Wall Rearing Behavior

Mice were placed individually in an acrylic box and were allowed to explore this box for 8 minutes. After this acclimation period, stereotyped behaviors such as on- and off-wall rearing were recorded. Rearing behavior was defined as any vertical movement that raised the mouse forepaws above the height of the mouse standing on four paws. On-wall rearing was recorded when mice touched the wall anytime during the incorporation. Rearing behavior had to be maintained for at least five seconds to be recorded. These criteria were based on a modification of the protocol by Russell et al. [34]. The analysis of these behaviors was done using the Observer software (version 3.0, Noldus, Wageningen, Netherlands), and the cumulative time spent on these behaviors was evaluated.

#### 2.4.3. Aggression Test (Intruder-Resident Paradigm)

In order to evaluate the presence of antagonist behavior due to As exposure, we followed a modified version of the resident-intruder test by Koolhaas et al. [35]. Briefly, a single mouse of each experimental group (control, 0.5 or 5.0 mg As/L in DW) remained in a neutral cage for 300 s; subsequently a male intruder was introduced into the resident's cage for 300 s. The confrontations were terminated after the first attack-bite; additional 300 s were added if no attack-bite by the resident occurred. The behavioral repertoire was videotaped and later analyzed using the Observer software. The events evaluated were latency to first attack, frequency and total duration of attacks, boxing, and tail rattling [36]. Encounters that included biting and that were at least three seconds apart were considered an attack [37].

### 2.5. Determination of DA, Serotonin, and Their Metabolites

DA, its metabolites 3,4-dihydroxyphenylacetic acid (DOPAC) and homovanillic acid (HVA), and serotonin (5-HT) were measured using HPLC with electrochemical detection as described elsewhere [20]. Briefly, a portion of striatum or frontal cortex was collected separately and disrupted by sonication in a solution of 0.1 M perchloric acid. The resulting homogenates were centrifuged at 10,000 ×g for 40 min, supernatants were frozen at −80°C, and pellets were digested in 0.5 M NaOH for protein determination by the Bradford technique. Briefly, a PerkinElmer pump series 200 (Waltham, MA, USA) was joined to a chromatographic column (Grace Davison Discovery Sciences, Deerfield, IL, USA) packed with a catecholamine adsorbosphere (3 *μ*m particle size, 100 × 4.8 mm). An electrochemical detector bioanalytical system, LC-4C (West Lafayette, IN, USA) was coupled to the system, the amperometric potential was set at 850 mV relative to the silver/silver chloride electrode, and the sensitivity of the detector was set at 5 (striatum) or 2 (frontal cortex) *η*A. The mobile phase consisted of 0.1 M monobasic phosphate solution containing 0.5 mM sodium octyl sulfate, 0.03 mM EDTA, and 13% (vol/vol) methanol. The results were analyzed with the TotalChrom Navigator version 6.3.1.0504 (PerkinElmer) and are expressed in ng/mg tissue protein. DA turnover was expressed as the ratio of DOPAC to DA, an index of DA utilization.

### 2.6. Analysis of mRNA Expression of DA Receptors by qPCR

Total RNA was isolated from striatum tissue samples using the TRIzol reagent (Invitrogen, Carlsbad, CA) and treated with RNase-free DNase (Promega, Madison, WI) to remove potential contamination by genomic DNA. RNA purity was determined from the ratio of absorbance readings at 260/280 nm with a NanoDrop ND-1000 (Thermo Scientific, Wilmington, DE, USA). Total RNA (0.5 *μ*g) from each sample was used for the cDNA synthesis using the M-MLV reverse transcriptase (Promega), Oligo dT (Invitrogen), and random hexamers primers following the manufacturer's instructions. Real-time PCR was performed with a LightCycler instrument version 1.5 (Roche, Mannheim, Germany) using the LightCycler FastStart DNA master SYBR Green I (Roche). The primers used in this experiment (Table 1) corresponded to DA receptor 1a (*Drd1*), DA receptor 2 (*Drd2*), and beta-actin (*Bact*). The cDNA samples previously prepared were diluted 1 : 5 and used as the template for the real-time PCR. Thermal conditions were 10 min denaturation, followed by 50 cycles at 95°C for 1 sec, 60°C for 10 sec, and 72°C for 12 sec. PCR amplifications were repeated in triplicate. At the end of each PCR reaction, a melting curve analysis was performed to confirm that a single product had been amplified. In addition, the expected size of the amplicon was verified by sequencing and electrophoresis of the PCR product in 1% EtBr agarose gels. Housekeeping gene *β*-actin was used as endogenous control of expression and the relative expression of the transcripts of each DA receptor of interest was calculated by using the 2^−ΔΔCt^ method [38].

### 2.7. Statistical Analysis

For the body weight gain and locomotor activity recorded for 24 hours, we used a two-way analysis of variance with repeated-measures in one factor (RMANOVA; treatment × time of day) followed by Fisher's LSD test in the case of significant main effects or interactions. Levels of 5-HT, DA, and their metabolites in brain regions were analyzed using one-way ANOVA with post hoc assessment in the event of main effects of treatment (Fisher's LSD tests). Data from aggression test,* Drd1* and* Drd2* mRNA levels, were analyzed using the Kruskall-Wallis test with Mann-Whitney *U* test as post hoc assessment in the event of main effects of treatment. The presence of body lesions was evaluated using chi-square test. Statistical significance was defined as *p* < 0.05

## 3. Results

### 3.1. Body Weight and General Appearance

**Figure 1:**
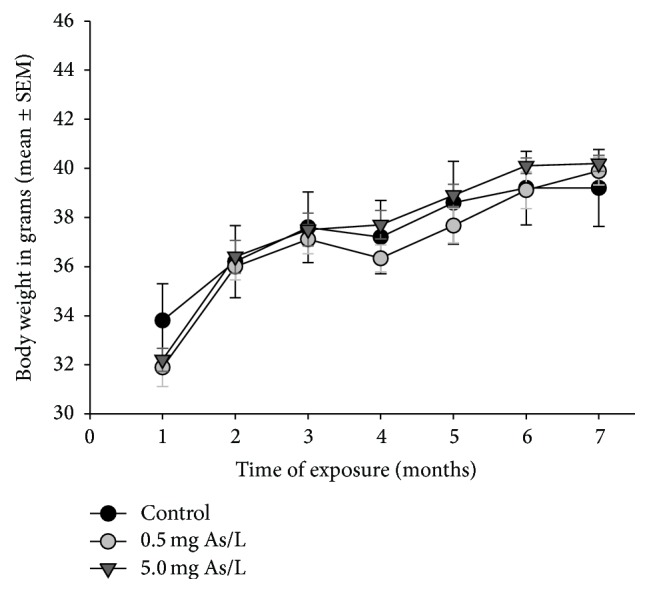
Growth rate of mice exposed to 0.5 or 5.0 mg As/L of drinking water for six months.

Mice exposed to 0.5 or 5.0 mg As/L of DW did not differ from control group in body weight evaluated monthly, although all groups gained weight overtime as shown in Figure 1.

Regarding the general appearance, As treatment did not increase the number of body lesions, or those changes not classified as lesions (whisker trimming, hair barbering, or disheveled coat) on mice treated with As. Mice treated with 5.0 mg As/L showed transitory increases in hair barbering at months 1 and 2 of As treatment, in comparison to control group.

### 3.2. Spontaneous Locomotor Activity

During the initial 1 h of recording, no significant effects of As treatment or interaction were found on horizontal activity, total distance, or stereotypy counts (data not shown).

**Figure 2:**
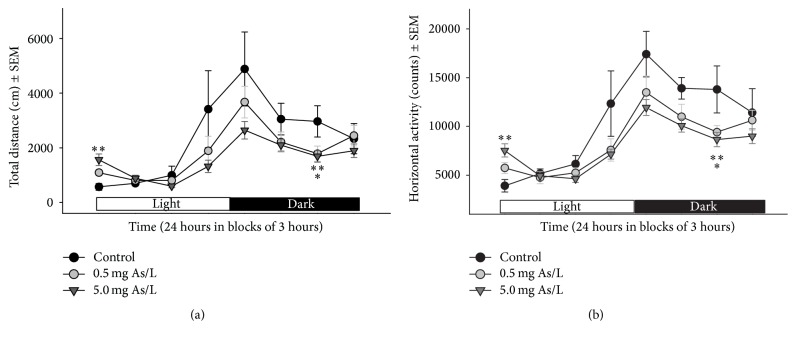
Effect of the chronic exposure to 0.5 or 5.0 mg As/L of drinking water on total distance (a) and horizontal activity (b). Spontaneous locomotor activity was recorded over the course of a 24-hour dark/light cycle at six months of As exposure. *∗* and *∗∗* denote differences between the 0.5 and 5.0 mg As/L groups, respectively, from the control group, *p* < 0.05.

No treatment effects were found from months 1 to 5 on 24-hour locomotor activity. Locomotor activity (total distance and horizontal activity) was significantly different between treated and control animals at six months of As exposure. For total distance, there was only a significant interaction (*F*(14,203) = 2.09, *p* = 0.0138) and sample effects (*F*(7,203) = 20.89, *p* < 0.0001). Post hoc analyses showed a biphasic effect of As treatment; mice treated with 5.0 mg As/L travelled less distance during the dark phase of the cycle but travelled more distance in comparison to the control group during the light phase of the dark-light cycle as shown in Figure 2. Regarding the horizontal activity, there was a significant effect of treatment (*F*(2,29) = 6.683, *p* = 0.0041), sample (*F*(7,203) = 28.736, *p* < 0.0001), and interaction (*F*(14,203) = 2.023, *p* = 0.0177). Post hoc analyses showed similar changes in both groups exposed to As, that is, hyperactivity at the beginning of the light part of the cycle and hypoactivity during the dark part of the cycle, as shown in Figure 2.

### 3.3. On- and Off-Wall Rearing Behavior

Regarding the on-wall rearing behavior, a significant effect of As treatment was observed at two and three months (*H*(2,33) = 6.166–8.569, *p* < 0.05) of As exposure. Post hoc analyses showed a higher frequency of on-wall rearing in the group treated with 5.0 mg As/L in comparison to the control group, on the second month of As exposure (*U* = 22.500, *p* = 0.0326). On the third month of As treatment, the group exposed to 0.5 mg As/L (*U* = 22.000, *p* = 0.0449) showed less frequency of on-wall rearing compared to the control group. The subsequent analyses at 4, 5, or 6 months of As treatment did not reveal differences in the frequency of this behavior (Table 2).

For the off-wall rearing behavior, there was a significant group effect (*H*(2,33) = 10.158, *p* = 0.0062) at one month of As exposure. Post hoc analyses showed that the group treated with 5.0 mg As/L displayed this behavior more than the control group (*U* = 22.000, *p* = 0.0298). Subsequent analyses at 2, 3, 4, 5, or 6 months of As treatment did not show any alteration in this behavior (Table 2).

### 3.4. Aggression Test (Intruder-Resident Paradigm)

At two months of exposure the group exposed to 5.0 mg As/L has shown decreases in the latency to attack and in tail rattling in comparison to control group. No more differences were found between As-treated and control group in the latency to first attack, frequency and total duration of attacks, boxing, and tail rattling during the six months of As treatment (Table 3).

### 3.5. Determination of DA and Its Metabolites and 5-HT

No significant As effects on the content of monoamines and their metabolites were found on striatum and frontal cortex of mice sacrificed after six months of As treatment, as shown in Table 4.

### 3.6. mRNA Levels of DA Receptors

**Figure 3:**
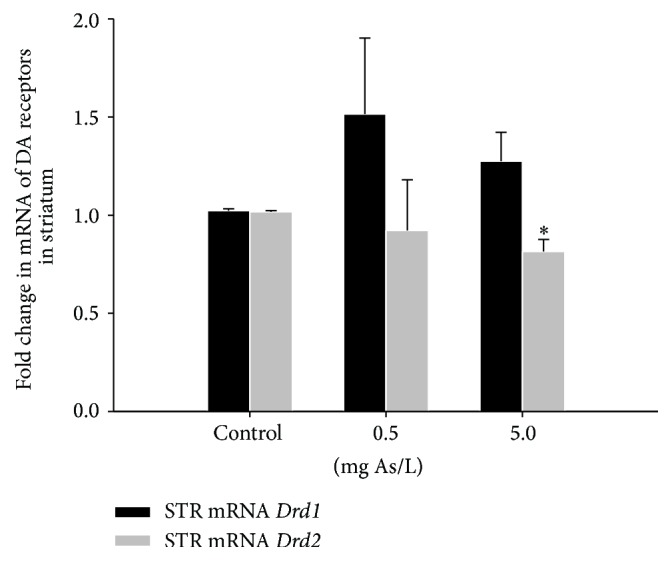
Dopamine receptors D1 (*Drd1*) and D2 (*Drd2*) mRNA expression in the striatum of male CD1 mice exposed chronically to 0.5 or 5.0 mg As/L of As in drinking water. Data was normalized to* Bact* and is presented as fold change relative to control group (mean ± SEM). *∗* denotes differences from the control group, *p* < 0.05.

Exposure to As caused a significant downregulation of* Drd2* mRNA in the striatum (*H* = (2, *N* = 24) = 6.180, *p* = 0.045) at the dose of 5.0 mg As/L (*U*'s = 8, *p* = 0.011) (Figure 3). In contrast, no significant changes on* Drd1* mRNA expression were observed in the striatum of groups treated with As in comparison to the control group (*H* = (2, *N* = 24) = 0.261, *p* = 0.878).

## 4. Discussion

From a toxicological perspective, there is considerable interest in finding essential biomarkers to evaluate the effects of environmental toxicants in the nervous system and in the dopaminergic neurotransmission system in particular. We found that chronic As exposure may alter targets other than the tissue levels of monoamines. In this regard, we found that chronic As exposure causes hypoactivity accompanied by decreases in mRNA expression of the* Drd2* receptor; we also found transitory and biphasic alterations on the on- and off-wall rearing behavior which could be due to transient alterations in the monoaminergic systems.

### 4.1. Body Weight and General Appearance

Chronic exposure to doses as low as 0.5 mg As/L or moderate doses such as 5.0 mg As/L of drinking water did not cause alterations in body weight compared to control group for the duration of the treatment which agrees with previous studies in rodents exposed to this metalloid [20, 28]. Previous studies of our group and others have demonstrated that in CD1 mice As enters and is distributed into brain regions after a short time exposure (9 days) [39, 40], and this is also observable in other chronic models [20, 28]. The transitory hairless patches found in the group exposed to 5.0 mg As/L at months 1 and 2 are in accordance with Nagaraja and Desiraju [41] who reported temporary hairless patches on male rats at day 20 of exposure to 5.0 mg As/kg BW as sodium arsenate, while Rodríguez et al. [42] also reported hair loss mainly during As exposure in the group of male rats treated with 20 mg As/kg BW.

### 4.2. As Exposure Produces Alterations in Locomotor Activity

The long-term exposure to As produces biphasic alterations in both total distance traveled and horizontal activity of the CD1 male mice locomotor activity. The alterations found in locomotor activity were not due to malaise by As exposure, since no changes in body weight or general appearance were found during As treatment.

Hypoactivity was found during the dark phase and hyperactivity was present at the beginning (initial 3 hours) of the light phase of the dark/light cycle on the group exposed to 5.0 mg As/L, while the group exposed to 0.5 mg As/L showed only the hypoactivity during the dark phase. These results are not in agreement with a previous study by Bardullas et al. [20] with the C57Bl/6J mouse strain where they reported that exposure to 0.5 mg As/L for 4 months produced hyperactivity during the light phase of the cycle, while no effects were reported in the group exposed to 5.0 mg As/L. The different responses to As exposure could be due to inherent variations between mice strains. Indeed, CD1 and C57BL/6J strains have been shown to differ in their susceptibility to the neurotoxin MPTP, the C57Bl/6J mouse strain being much more susceptible [43] since this strain has less midbrain DA neurons [44] and lower DA function in striatum [45]. On the other hand for hyperbaric oxygen-induced convulsions, the CD1 strain is more sensitive [46]. The same group reported increased striatal norepinephrine levels in CD1 in comparison to the C57Bl/6J mouse strain [47]. At the neuroanatomical level, the C57Bl/6J mice have larger cerebral cortex and ventricular compartments than age-matched CD1 mice, but the volume of the striatum is bigger in the CD1 strain [48].

Decreases in motor activity due to As exposure have already been shown in studies using rats [19, 28, 42, 49]. In these studies the decreases in locomotor activity were found when doses above 10 mg As/L were used. It is important to mention that the pattern of hypoactivity due to the exposure of 0.5 or 5.0 mg As/L found in this study is similar to the one found by Rodríguez et al. [28] using male rats treated with the high dose of 50 mg As/L for twelve months. These observations suggest that male CD1 mice may be more sensitive to As intoxication in comparison to the male Sprague-Dawley rat that needs doses as high as 50 mg As/L of DW but less sensitive than the C57Bl/6J male mice which need only four months of exposure to show changes in locomotor activity [20].

### 4.3. On- and Off-Wall Rearing Behavior

The transitory and biphasic alterations observed at months 1 and 2 of As treatment on the on- and off-wall rearing behavior could be due to alterations in the monoaminergic systems. It has been shown that the administration of amphetamine to adult male rats increases both on- and off-wall rearing [34] whereas the lesion of noradrenergic or dopaminergic systems decreases rearing in mice [50]. In the present study we verified the content of monoamines only at the end of the six months of As treatment.

### 4.4. Aggressive Behavior

The protocol and doses of As exposure used in this study do not evidence increased aggressive behavior in CD1 male mice. The As-treated mice only showed aggressive behavioral components necessary to establish a social hierarchy inside a group, such as grooming, chasing, and barbering [51].

### 4.5. Monoamine Levels

Chronic As exposure did not cause alterations in monoamine content in striatum or prefrontal cortex of CD1 male mice. This explains in part the lack of aggressive behavior in mice exposed to As. The absence of alterations in brain monoamine levels in this study is in agreement with a previous study that reported no changes in DA or its metabolites in striatum of C57Bl/6J male mice exposed to similar doses of As used in this study [20].

According to several studies As exposure causes alterations in several neurotransmitter systems including the monoaminergic systems only when rodents are exposed to high doses of this metalloid [19–21, 28, 41, 42, 52]. In addition to the differences in As doses, the discrepancies between this study and those present in the literature could be due to differences in the species or strain used, the duration of treatments, the mode of administration, and the source of As (sodium arsenite, sodium arsenate, or arsenic trioxide).

Earlier studies had suggested that hypoactivity could be due to alterations in the dopaminergic system [53], but from the data presented here we can conclude that the hypoactivity observed in these rodents after six months of As exposure is not due to alterations in brain monoamine levels. Our finding however does not discard the involvement of changes at the level of dopaminergic signaling, DA release, or DA receptors, as we discuss below, since it is well known that As can stimulate or inhibit several signaling routes [54, 55] or affect different levels of DA system regulation which could be involved in movement control.

### 4.6. mRNA of DA Receptors Drd1 and Drd2

In this study, we found that only the* Drd2* was downregulated in the striatum of mice chronically exposed to 5.0 mg As/L; this finding is important because it involves changes at postsynaptic level. Whereas activation of presynaptic DA D2 receptors generally causes a decrease in DA release that in turn results in decreased locomotor activity, activation of postsynaptic receptors stimulates locomotion [56]. This result could explain in part why we observed hypoactivity in mice exposed to 5.0 mg As/L and agrees with a previous study in which mRNA expression of* Drd2* was observed to be downregulated in a dose-dependent manner by chronic exposure to As in the nucleus accumbens of the rat [28]. It must be noted that other studies have shown the opposite effect in mRNA expression of D2 in the striatum of rats postnatally exposed to 2 or 4 mg As/kg of BW [29] and in striatum of mice exposed to 1–100 mg As/L DW for three weeks [57].

The fact that in this study mice presented the above stated alterations at six months of As exposure is particularly relevant, since similar alterations were developed only by male Sprague-Dawley rats treated with the high dose of 50 mg As/L and only after one year of treatment. Locomotion is primarily controlled by the ventral striatum through activation of Drd1 and Drd2 and Drd3 receptors [58]; and synergistic interactions are necessary to produce complete locomotor stimulation [59]. The finding of downregulation of* Drd2* in striatum in this study is highly relevant since mutant animals lacking Drd2 are akinetic and bradykinetic, with significantly reduced spontaneous movement that resembles the extrapyramidal symptoms of Parkinson's disease [60]. In the present study, we must be cautious with the interpretation of the downregulation of* Drd2*, because the locomotor hypokinesia was observed only in the dark phase and appears not to be a constant condition. But we emphasize that it is possible that the locomotor hypoactivity seen in the mice exposed to As may be the result of downregulation of striatal* Drd2*.

It remains a challenge to correlate the changes in behavior with neurochemical alterations or disruptions in expression of genes related to the dopaminergic system from a classical toxicological point of view. Moreover, in the case of DA receptors, their specific participation in behavioral paradigms is still a matter of debate. Alternative explanations to our results in relation to DA content and* Drd2* expression in chronic As exposure must consider the complexity of the dopaminergic system. DA receptors are regulated at several levels including transcription and synthesis, internalization and transport, and changes in affinity.

As mentioned earlier, chronic As exposure has been shown to change DA neurochemistry as evidenced by gender-specific alterations in locomotor activity and molecular alterations related to tyrosine hydroxylase (TH) and antioxidant mRNA expression in mice [20], changes in mRNA expression of DA receptors D1 (*Drd1*) and D2 (*Drd2*) in striatum and nucleus accumbens of male rats [28], increased mRNA expression of DA receptors D1 (*Drd1*), D2 (*Drd2*), D3 (*Drd3*), and D4 (*Drd4*) together with decreased TH in striatum and cerebral cortex of adult C57Bl/6 mice [57], increased binding and mRNA expression of D2 receptors, and increased expression of TH protein levels in striatum of Wistar rats [29].

DA receptors regulate the expression of their own genes; for instance, disruption of the nigrostriatal dopaminergic pathway with 6-OHDA increases* Drd2* mRNA expression in striatonigral and striatopallidal neurons [61]. Similarly, chronic treatment with haloperidol increases* Drd2* mRNA expression in the caudate putamen [62]. In this study, it is possible that As exposure impaired DA receptor-dependent signaling resulting in* Drd2* mRNA downregulation. Another possibility is that dopaminergic transmission could have been increased by As treatment. This would not necessarily depend on the de novo synthesis of DA but on alterations in its transporter, as is the case of mice lacking dopamine transporter (DAT). These mice show overactive dopaminergic transmission and downregulation in the mRNA of* Drd1* and* Drd2* [63]. Further studies are necessary to evaluate this hypothesis in the model of chronic exposure to As.

In conclusion, exposure to 0.5 or 5.0 mg As/L for six months in CD1 male mice did not alter the typical intraspecific behaviors necessary to establish a social hierarchy, nor did it alter normal monoamine levels. Mice treated with the highest dose of As displayed hypoactivity and decreases in* Drd2* mRNA in the striatum at the end of treatment. The fact that these changes were detected after six months of As exposure is of particular relevance, since similar alterations were observed in male Sprague-Dawley rats treated with a high dose of As (50 mg As/L) only after one year of treatment. Based on these findings, we can conclude that the CD1 male mouse is more sensitive to As exposure than the Sprague-Dawley male rat and may represent a better model of As neurotoxicity. Although the interpretation and prediction of the effects of environmental toxicants on the dopaminergic system remain a complex issue, from a toxicological perspective the contribution of this study is the hypothesis that DA receptors, particularly* Drd2*, may be a potential target for the effects of As exposure in rodents.

## Figures and Tables

**Table 1:** Primer sets used for SYBR green-based qPCR analysis of dopamine receptors.

Target	Abbreviation	Primer	Primer sequence	Primer length (nt)	Amplicon size (bp)	Gene bank accession number
Dopamine receptor D1a	*Drd1*	Forward	5′ CAG TCC ATG CCA AGA ATT GCC AGA 3′	24	255	NM_010076.3
		Reverse	5′ AAT CGA TGC AGA ATG GCT GGG TCT 3′	24		

Dopamine receptor D2	*Drd2*	Forward	5′ TGA ACA GGC GGA GAA TGG 3′	18	70	NM_010077.2
		Reverse	5′ CTG GTG CTT GAC AGC ATC TC 3′	20		

Beta-actin	*Bact*	Forward	5′ CCA GGT CAT CAC TAT TGG CAA CGA G 3′	25	141	NM_007393.3
		Reverse	5′ TCT TTA CGG ATG TCA ACG TCA CAC T 3′	25		

nt: nucleotide, bp: base pair.

**Table 2:** On- and off-wall rearing behavior.

Time of exposure	Control	0.5 mg As/L	5.0 mg As/L
	On-wall rearing		
Month 1	2.50 (6.00)	3.50 (8.00)	7.00 (7.50)
Month 2	2.50 (3.50)	1.50 (5.50)	**5.00 **(5.50)^*∗*^
Month 3	5.00 (6.00)	**0 **(2.50)^*∗*^	7.00 (11.50)
Month 4	5.00 (7.50)	5.50 (7.50)	4.00 (3.50)
Month 5	0.50 (7.50)	5.50 (10.50)	7.00 (7.75)
Month 6	3.00 (9.00)	3.50 (4.00)	6.00 (10.25)

	Off-wall rearing		
Month 1	1.50 (7.00)	3.00 (2.50)	**10.00 **(6.75)^*∗*^
Month 2	7.50 (7.50)	1.00 (4.50)	8.00 (9.75)
Month 3	4.00 (6.50)	0.50 (3.50)	8.00 (11.25)
Month 4	3.50 (7.50)	1.50 (6.00)	4.00 (5.00)
Month 5	3.00 (8.00)	2.50 (8.50)	3.00 (9.75)
Month 6	4.00 (9.00)	3.00 (4.00)	8.00 (11.00)

Values are median and interquartile range of cumulative spent time on these behaviors (*n* = 7–13).

*∗* denotes differences from the control group, *p* < 0.05.

**Table 3:** Aggression test (intruder-resident paradigm).

Time of exposure	Control	0.5 mg As/L	5.0 mg As/L
	Latency to first attack (time)		
Month 1	382.25 (554.90)	600.00 (487.60)	600.00 (378.02)
Month 2	154.80 (538.55)	446.10 (447.10)	**600.00 (0.00)**
Month 3	178.75 (401.35)	600.00 (473.85)	600.00 (216.30)
Month 4	600.00 (283.00)	450.00 (503.90)	516.20 (371.32)
Month 5	600.00 (424.50)	600.00 (311.40)	600.00 (297.10)
Month 6	600.00 (294.55)	600.00 (419.75)	600.00 (133.27)

	Frequency (counts)		
Month 1	1.00 (2.50)	0 (1.50)	0 (1.00)
Month 2	1.00 (2.00)	0.50 (2.50)	0 (0)
Month 3	1.00 (0.50)	0 (1.50)	0 (1.00)
Month 4	0 (0.50)	0.50 (1.50)	1.00 (1.00)
Month 5	0 (1.50)	0 (1.00)	0 (1.25)
Month 6	0 (0.50)	0 (1.0)	0 (1.25)

	Total duration of attacks (time)		
Month 1	23.80 (1213.15)	0 (22.85)	0 (22.49)
Month 2	32.20 (46.20)	6.70 (99.50)	0 (0)
Month 3	11.75 (30.25)	0 (57.40)	0 (5.24)
Month 4	0 (6.80)	5.6 (22.60)	0.76 (22.37)
Month 5	0 (12.25)	0 (11.45)	0 (13.40)
Month 6	0 (8.30)	0 (11.35)	0 (3.17)

	Boxing (counts)		
Month 1	0 (0)	0 (2.00)	0 (1.00)
Month 2	0 (1.00)	0 (0.50)	0 (0)
Month 3	0.50 (1.00)	0 (1.00)	0 (0.25)
Month 4	0 (0)	0 (0.50)	0 (1.25)
Month 5	0 (0)	0.50 (1.00)	0 (0.25)
Month 6	0 (1.50)	0.50 (1.50)	0 (0)

	Tail rattling (counts)		
Month 1	0 (9.00)	0 (3.00)	0 (1.00)
Month 2	1.00 (9.50)	0.50 (4.00)	**0 (0)**
Month 3	4.50 (10.00)	0 (2.50)	0 (0.50)
Month 4	0 (1.50)	1.00 (3.00)	1.00 (3.25)
Month 5	0 (11.00)	0.50 (4.00)	0 (2.75)
Month 6	0 (8.00)	1.50 (9.50)	0 (2.00)

Values are median and interquartile range (*n* = 7–13).

**Table 4:** Regional brain content of monoamines (ng/mg protein) in striatum and frontal cortex of mice exposed to 0.5 or 5.0 mg As/L of drinking water for six months.

Brain region	Treatment	DA	DOPAC	HVA	5-HT	5-HIAA	DOPAC/DA
Striatum	Control	80.60 ± 13.00	5.29 ± 1.56	4.22 ± 0.95	5.71 ± 1.26	2.87 ± 0.57	0.06 ± 0.01
	0.5 mg As/L	91.22 ± 19.37	2.86 ± 0.04	4.20 ± 0.70	5.19 ± 1.36	2.58 ± 0.48	0.06 ± 0.01
	5.0 mg As/L	55.70 ± 6.83	3.70 ± 0.83	3.35 ± 0.46	5.80 ± 1.05	2.29 ± 0.35	0.07 ± 0.01

Frontal cortex	Control	1.17 ± 0.31	ND	ND	4.37 ± 1.51	2.42 ± 0.72	—
	0.5 mg As/L	1.86 ± 0.39	ND	ND	6.18 ± 1.16	1.88 ± 0.42	—
	5.0 mg As/L	1.53 ± 0.23	ND	ND	8.06 ± 2.17	2.22 ± 0.35	—

Values are mean ± SEM (*n* = 7–13) and are reported as ng/mg of protein. DA, dopamine; DOPAC, 3,4-dihydroxyphenylacetic acid; HVA, homovanillic acid; 5-HT, serotonin; 5-HIAA, 5-hydroxyindoleacetic acid. ND: not detected.
